# The Role of 24-Hour Holter Electrocardiogram in the Early Detection of Atrial Fibrillation in Newly Diagnosed Acute Ischemic Stroke Patients

**DOI:** 10.7759/cureus.62566

**Published:** 2024-06-17

**Authors:** Wijdan B Alriyami, Muhammad A Sadiq, Mohamed Al Rawahi, Sheeraz Ahmed, Fahad A Kindi, Muhammad A Khatri

**Affiliations:** 1 Medicine, Sultan Qaboos University, Muscat, OMN; 2 Medicine, Oman Medical Specialty Board, Muscat, OMN

**Keywords:** pvcs as predictive marker, atrial fibrillation detection, holter attachment delay, holter monitoring efficacy, ischemic stroke

## Abstract

Introduction

Stroke is a leading cause of death and disability globally, with atrial fibrillation (AF) recognized as a significant risk factor due to its association with increased stroke recurrence and mortality. Timely detection of AF is crucial to prevent recurrent strokes and improve outcomes. This study primarily aimed to evaluate the utility of 24-hour Holter monitoring for AF detection in acute ischemic stroke patients.

Methods

This retrospective observational study examined data from 207 patients admitted with acute ischemic stroke to a tertiary-care hospital over a two-year period. Patients with pre-existing AF, transient ischemic attacks, unconfirmed diagnoses, and missing Holter reports were excluded. A total of 140 patients were included in the analysis. The study investigated AF detection rates, the relationship between AF and stroke risk factors, other Holter findings, and the time delay in attaching Holter monitors.

Results

Of the 140 patients evaluated, AF was detected in nine (6.4%), exclusively in those aged ≥65 years. The most prevalent risk factors among the study participants were hypertension (74.3%) and diabetes (61.4%). No significant correlations were observed between AF and the analyzed stroke risk factors. The median delay for Holter device attachment was 3,503 minutes (approximately two days and 10 hours), with longer delays noted in males (4,084 mins (approximately two days and 20 hours) vs. 2,565 mins (approximately one day and 18 hours), p=0.005). Premature atrial complexes (PACs) were notably associated with the absence of AF, suggesting their potential role as markers for undiagnosed AF.

Conclusion

The study highlights the limited utility of 24-hour Holter monitoring in detecting AF in acute ischemic stroke patients, advocating for extended monitoring durations, especially in older patients. To improve AF detection, potential strategies include using longer monitoring periods and optimizing hospital workflows to reduce delays in attaching Holter devices. These approaches can minimize the risk of underdiagnosing paroxysmal AF, thereby preventing recurrent strokes and improving patient outcomes. Further investigation into PACs as potential predictive markers for AF is warranted.

## Introduction

Stroke represents a significant global health issue, being a leading cause of death and disability worldwide [1]. Ischemic stroke, which primarily results from thrombotic or embolic occlusions of a cerebral artery, accounts for the majority of stroke cases in the United States, leading to substantial healthcare costs [1,2].

Several common risk factors contribute to the incidence of stroke, including hypertension, atrial fibrillation (AF), hypercholesterolemia, coronary artery disease, diabetes, smoking, and a sedentary lifestyle [2]. Aging also plays a significant role. Notably, AF is an independent risk factor that increases the risk of stroke fivefold [3]. In individuals over 75 years old, AF is the leading cause of stroke, accounting for 15% of cases [4]. AF occurs 30% more frequently in this population compared to those without AF, consequently leading to increased mortality rates. The paroxysmal nature of AF attacks poses a significant challenge for medical assessment, as clinicians often miss the diagnosis due to the asymptomatic presentation and the typically short monitoring period post-stroke, usually limited to 24 hours [1].

International guidelines recommend cardiac monitoring with telemetry for at least the first 24 hours in adults with ischemic stroke (class I) [4]. However, prolonged cardiac monitoring during hospitalization has been shown to increase AF detection in patients with stroke or transient ischemic attack (TIA). Monitoring for 72 hours significantly improves AF detection by 2.6-fold compared to 24-hour Holter monitoring [1]. The Cryptogenic Stroke and Underlying AF (CRYSTAL AF) trial demonstrated that 24-hour Holter monitoring had the lowest AF detection rate (1.3%), while a 30-day event recorder achieved the highest detection rate (22.8%) [5].

In hospitals not equipped with telemetry systems, 24-hour Holter monitoring is used for the initial screening of AF in acute ischemic stroke patients. Extending monitoring beyond 24 hours significantly increases the probability of detecting silent tachyarrhythmia episodes [6]. A prospective multicenter study indicated that extending the duration of Holter monitoring from 24 to 72 hours significantly increases AF detection rates in stroke survivors. Despite these findings, there is no consensus on the optimal monitoring methods and durations [6]. Additionally, considerable delays in attaching 24-hour Holter devices can occur, depending on whether stroke patients are admitted during the day or night, potentially leading to missed cases and an underestimation of AF prevalence.

This study primarily aimed to evaluate the utility of 24-hour Holter monitoring in acute ischemic stroke patients and to calculate the time interval (delay) of attaching Holter monitoring devices to stroke patients after admission. The study seeks to address the identified gaps in knowledge by providing insights into the clinical implications of underdiagnosing AF and emphasizing the importance of improving AF detection methods to prevent recurrent strokes and reduce mortality rates.

## Materials and methods

This retrospective observational study utilized data from the Hospital Information System (HIS) at a tertiary-care hospital, employing the electronic medical record system. The investigation focused on assessing 24-hour Holter records of patients admitted with acute ischemic stroke over a two-year period.

Inclusion criteria

The study included consecutive patients who were older than 18 years and had been admitted with an acute ischemic stroke during a specified two-year study period. Stroke diagnoses were confirmed by using established protocols, which included imaging criteria such as MRI or CT scans. This criterion was designed to focus on adults who experienced a recent stroke, ensuring that the data analyzed was relevant to acute care and immediate cardiac monitoring needs.

Exclusion criteria

Several criteria were established for excluding patients from the study. These included individuals with pre-existing AF, those who had suffered a TIA, unconfirmed cases (patients whose diagnosis of acute ischemic stroke could not be definitively established based on available medical records and imaging studies), and those with missing complete Holter reports (instances where the Holter monitoring data was incomplete or unavailable for the full 24-hour period required for analysis). After the initial evaluation of 207 patients, 67 were excluded based on these criteria, leaving 140 patients in the final study cohort.

Data collection

The information about gender, age, risk factors of stroke, and Holter findings were collected through the detailed chart reviews of patients' electronic medical records. This study was approved by the Medical Research Ethics Committee (MREC) of the College of Medicine & Health Sciences, Sultan Qaboos University (Approval No. MREC #2470). All patient data were anonymized to ensure confidentiality. The date and time of stroke diagnosis and Holter monitoring were obtained to calculate the delay of Holter device attachment in minutes. Risk factors of stroke, such as hypertension, diabetes mellitus, previous stroke, ischemic heart disease (IHD), smoking, alcohol intake, atherosclerosis, heart failure, and dilated cardiomyopathy, were obtained from the clinical notes. The type of Holter device used for all patients was the Getmed, CardioMem® CM 4000, Germany. Holter results were interpreted based on standard criteria. Several Holter outcomes were reported, including the presence of AF, atrial tachycardia (AT), premature atrial complexes (PACs), premature ventricular complexes (PVCs), ventricular and supraventricular tachycardia, heart blocks, and sinus pauses. Specific types of arrhythmias assessed during Holter monitoring included non-sustained (lasting less than 30 seconds) atrial and ventricular tachycardias.

Statistical analysis

Collected data were analyzed using Statistical Package for the Social Sciences (IBM SPSS Statistics for Windows, IBM Corp., Version 28.0, Armonk, NY). The frequency of those detected to have AF was calculated and represented for gender and meaningful age groups. The time between stroke diagnoses and Holter device attachment was recorded in minutes. Continuous variables were tested for normality of distribution using the Kolmogorov-Smirnov test and presented as median (IQR) and mean (standard deviation) as appropriate. The non-normal variables were tested by the Mann-Whitney U test. The categorical variables were tested for their association with AF using Chi-square and Fisher’s test. Descriptive data of the demographical findings were obtained. P-values <0.05 were considered to be significant.

## Results

The data of 207 patients were evaluated, and 67 (32.37%) patients were excluded due to pre-existing AF, hemorrhagic stroke, TIA, and an unconfirmed diagnosis of ischemic stroke. The remaining 140 (67.63%) were eligible for the study. Eighty-seven (62.1%) patients were males. The mean age was 66.51 years (SD ± 12.563), ranging from 36 to 95 years old.

From the total sample size, 104 individuals (74.3%) had hypertension, 86 (61.4%) had diabetes, 40 (28.6%) had a previous history of stroke, and 26 (18.6%) had a history of IHD (Table 1). However, there were no statistically significant associations found between AF and the listed stroke risk factors (Table 2). This indicates that while these conditions are common among stroke patients, they did not show a significant correlation with AF detection in our study cohort.

**Table 1:** The distribution of stroke risk factors among the study sample

Stroke risk factors, n (%)	Total n = 140
Hypertension	104 (74.29%)
Diabetes mellitus	86 (61.43%)
Previous strokes	40 (28.6%)
Ischemic heart disease	26 (18.57%)
Smoking	16 (11.43%)
Alcoholism	7 (5%)
Atherosclerosis	1 (0.71%)
Dilated cardiomyopathy	3 (2.14%)

**Table 2:** Demographical features and Holter findings in atrial fibrillation (AF) and no AF patients

Characteristic features	No AF	AF	p-value
Stroke risk factors, n (%)			
Hypertension	99 (95.2%)	5 (4.8%)	0.235
Diabetes mellitus	81 (94.2%)	5 (5.8%)	0.734
Previous strokes	36 (90.0%)	4 (10.0%)	0.276
Ischemic heart disease	23 (88.5%)	3 (11.5%)	0.368
Smoking	15 (93.8%)	1 (6.3%)	1
Alcoholism	7 (100%)	0 (0%)	1
Atherosclerosis	0 (0%)	1 (100%)	0.067
Dilated cardiomyopathy	3 (100%)	0 (0%)	1

Only 9 out of 140 acute ischemic stroke patients were detected to have AF using 24-hour Holter monitoring, resulting in a 6.4% detection rate. AF was detected solely in patients aged ≥65 years (100%), which accounted for 11.54% of AF detection in this age group (p=0.005). This significant p-value indicates that age ≥65 years is strongly associated with AF detection in our study population. AF was observed more frequently in males than in females; however, this difference was not statistically significant. The delay time, measured in minutes, was calculated from the time of stroke diagnosis to the time of Holter device attachment. The median delay time was 3,503 minutes (approximately two days and 10 hours), with a range from 57 to 158,249 minutes. The median delay was higher in males than in females, being 4,084 minutes (approximately two days and 20 hours) and 2,565 minutes (approximately one day and 19 hours), respectively (p=0.005) (Table 3). Table 3 also states the median (IQR) in minutes for gender, age categories, and AF/no AF as follows: males 4,084 (3,363); females 2,565 (3,437); age <65 years 3,718 (3,787); age >65 years 3,357 (3,231); AF not present 3,740 (3,587); AF present 2,678 (2,272).

**Table 3:** Holter attachment delay time differences among atrial fibrillation (AF) and no AF patients, gender, and age groups Note: * indicates a statistically significant value (p<0.05).

Categories		Median delay (minutes) (median, IQR)	p-value
Gender	Males	4,084 (3,363)	0.005*
	Females	2,565 (3,437)	
Age	≤65 years	3,357 (3,787)	0.88
	>65 years	3,718 (3,231)	
AF presence	AF patients	2,678 (3,587)	0.07
	No AF patients	3,740 (2,272)	

The association of AF with Holter findings, including PACs, PVCs, sinus pauses, heart blocks, AT, and ventricular and supraventricular arrhythmias was obtained (Table 4). PACs were found to be more prevalent in no AF patients compared to AF patients (98.9% vs. 1.1%, p=0.001). The other Holter findings were not significant between the two groups.

**Table 4:** Comparison of Holter findings in atrial fibrillation (AF) and no-AF patients Note: * indicates non-sustained ventricular and atrial tachycardias.

Holter findings, n (%)	No AF	AF	p-value
Premature ventricular complexes	106 (93.0%)	9 (7.0%)	1
Premature atrial complexes	92 (98.9%)	1 (1.1%)	0.001
Sinus pauses	1 (100%)	0 (0%)	1
Heart blocks	3 (100%)	0 (0%)	1
Atrial tachycardia	25 (96.2%)	1 (3.8%)	1
Other arrhythmias*	16 (94.1%)	1 (5.9%)	1

## Discussion

Despite current guidelines advocating for at least 24 hours of continuous monitoring in acute ischemic stroke cases, there is no consensus on the optimal monitoring methods and durations. In our study, AF was detected in 6.4% of participants, all over 65 years, using 24-hour Holter monitoring. The detection of AF was nearly similar across genders, aligning with results from other studies that reported detection rates ranging from 0 to 10% [7]. The detection rate in our study was slightly higher than that in a prospective multicenter cohort study, where the rate was 2.6% [7]. This could be due to our study being conducted in a single tertiary-care referral center. Conversely, another similar prospective study utilizing 24-hour Holter monitoring reported a high detection rate of 25.2% [8]. This significant difference might be due to a non-homogeneous distribution of sample size in their study, with the majority of the population being elderly. The gender distribution of our results was analogous to that reported by Grond et al. and Göksu et al. [7,9], whereas Goel et al. observed significantly higher detection rates among males than females, which contrasts with our findings but is consistent with our age-related results [8]. These differences could be attributable to the overall gender distribution in the study sample. It is noteworthy that AF incidence is higher in the population over the age of 65 years.

The detection rate using 24-hour Holter is considered to be low when compared with the expected AF in acute ischemic stroke patients. Various studies have shown that extending the monitoring duration to 72 hours significantly increases the AF detection rate among this group. Grond et al. reported that prolonging the monitoring period from 24 to 72 hours improved the detection rate from 2.6% to 4.3% [7]. Furthermore, employing 14-day event Holter monitoring substantially enhanced AF detection to 14.6% [10].

The relatively low detection rate in our study may not represent the actual AF patients in our sample because of the low sensitivity of the 24-hour Holter monitoring compared with other modalities, such as long-term cardiac monitoring, external loop recorders (ELR), internal loop recorders (ILR), and mobile cardiac outpatient telemetry (MCOT) [7]. Two large-scale randomized controlled trials have demonstrated that ambulatory ECG monitoring with a 30-day event-triggered recorder and implantable cardiac monitors significantly enhance the detection of AF, thus facilitating the timely initiation of anticoagulation therapy [11,12]. Furthermore, studies suggest that loop recorders offer a superior diagnostic yield compared to seven-day ambulatory Holter monitoring [13]. The improved detection rates provided by these advanced devices over conventional 24-hour ECG and seven-day Holter monitoring enable more patients to receive early and appropriate treatment interventions. Early administration of anticoagulants, such as warfarin and direct oral anticoagulants (DOAC), has been shown to reduce the incidence of stroke by 40%, proving more effective than antiplatelet therapy [14]. Ensuring accurate AF diagnosis in stroke patients is crucial for enabling these life-saving therapeutic interventions.

Early detection of AF cases could prevent future recurrent strokes, as the stroke recurrence rate in AF patients is 30% higher than in those without AF. This not only results in worse neurological recovery but also leads to increased length of hospital stay, higher total medical care costs, and greater mortality [6,15,16]. However, timely detection remains challenging, as evidenced by the median delay of two days and 10 hours in attaching Holter devices to our patients from the time of diagnosis. This delay may compromise the effectiveness of interventions aimed at reducing the risks associated with AF. The median delay in attaching Holter devices was significantly longer in males as compared to females. However, no such difference was observed among detected AF cases across age groups. Jabaudon et al. have observed a large difference where the median delay time was eight days [17]. It is possible that such delay times could be attributed to their study being conducted in a primary care center with potentially limited availability of Holter devices, as opposed to tertiary care centers where such devices are more readily accessible. Conversely, other studies have documented a mere 5.5-hour delay, starkly contrasting with the 58 hours observed in our study center [18]. These differences may stem from our center’s restricted access to Holter devices and inconsistencies in admission times during the day and night, potentially leading to prolonged delays for patients admitted late at night. Such delays risk missing critical cases of paroxysmal AF, which are predominantly silent. Another study found that patients with no AF had a significant delay of 0.43 days or almost 10 hours [7]. Our study found no significant association between these two variables. The relatively small sample size could explain why such associations were not found to be significant. Our results revealed a gender disparity, with males experiencing more delays in Holter device attachment, though the reasons for this remain unclear.

Longer delays in Holter device attachment can potentially impact AF detection rates and patient outcomes, as timely monitoring is crucial for accurate diagnosis. The significant delay in males may suggest differences in clinical workflow or patient presentation times that warrant further investigation. Addressing these delays could improve AF detection rates and subsequently enhance patient outcomes by allowing for timely intervention.

Several stroke risk factors were tested for their associations with AF, but none of the studied risk factors were found to be significant (Table 2). Furthermore, the analysis extended to other Holter monitoring findings, revealing that the presence of PACs was significantly associated with non-AF cases, demonstrating a 98.9% prevalence compared to 1.1% in AF cases (p=0.001). The study found that supraventricular ectopic activity, such as supraventricular extrasystoles, runs, and pairs of supraventricular extrasystoles, was significantly higher in cryptogenic ischemic stroke patients with newly diagnosed AF during prolonged 14-day Holter event monitoring [10]. A systematic review and meta-analysis have shown that frequent PACs on 24-48-hour Holter monitors are significantly associated with AF in older patients without a history of the condition [19]. This finding suggests that PACs might serve as early indicators of undiagnosed AF, potentially identifying a significant number of patients who remain undetected and, thus, are at increased risk.

Limitations

The study utilized 24-hour Holter monitoring to detect AF in acute ischemic stroke patients, focusing primarily on an elderly population over the age of 65. While our findings suggest potential benefits in extending monitoring duration to improve AF detection rates, several limitations must be considered.

The study's retrospective nature introduces inherent methodological constraints that could influence the findings. Specifically, our analysis is based on data collected from a single tertiary-care center, which may not represent broader patient populations or settings. This limitation may impact the generalizability of our results and could introduce selection bias, as patient demographics and healthcare practices may vary across different settings. The sample size, although adequate for initial observations, may not be sufficient to generalize results across diverse populations or to detect smaller but clinically relevant effects. The limited sample size may also increase the risk of type II error, where true effects are not detected due to insufficient statistical power.

Furthermore, our study did not include a control group for comparison, which limits our ability to assess the relative utility or effectiveness of 24-hour Holter monitoring against other monitoring methods. The retrospective design also meant that we were unable to track the reasons for delays in Holter attachment, which could provide insights into improving clinical practice.

These factors underscore the need for larger, multicenter prospective studies to validate our findings. Future research should aim to include more diverse patient populations, spanning different age groups and stroke severities, to better understand the broader implications of extended monitoring durations for AF detection in acute ischemic stroke patients. Prospective studies can also address potential confounding factors more effectively and provide stronger evidence for clinical decision-making in stroke management.

## Conclusions

In conclusion, our study underscores the limitations of 24-hour Holter monitoring in detecting AF among acute ischemic stroke patients, advocating for extended monitoring periods to improve detection rates, particularly in patients aged 65 years or older. The notable delay in attaching Holter devices, which may risk missing instances of paroxysmal AF, alongside significant gender disparities, highlights the need for optimized protocols to expedite the initiation of monitoring.

Additionally, while the association of PACs with patients without AF is intriguing, further research is needed to validate their potential as predictive markers for undiagnosed or future AF. Future research should aim to broaden the sample size and explore diverse monitoring approaches. Validating these insights and refining patient care strategies requires longer monitoring of patients. We encourage collaboration among researchers, clinicians, and healthcare policymakers to implement evidence-based interventions and improve patient outcomes.
